# Synbiotic Nutraceutical Mitigates Gestational Diabetes Effects and Cardiovascular Dysfunction in Rat Offspring

**DOI:** 10.1002/mnfr.70340

**Published:** 2025-11-28

**Authors:** Deborah Emanuelle de Albuquerque Lemos, Evandro Leite de Souza, Micaelle Oliveira de Luna Freire, Jaielison Yandro Pereira da Silva, Paulo César Trindade da Costa, Josiane de Campos Cruz, Alisson Macário de Oliveira, João Felipe Mota, José Luiz de Brito Alves

**Affiliations:** ^1^ Department of Nutrition Health Sciences Center Federal University of Paraíba João Pessoa Paraíba Brazil; ^2^ Biotechnology Center Federal University of Paraíba João Pessoa Paraíba Brazil; ^3^ Center For Biological and Health Sciences State University of Paraíba Campina Grande Paraíba Brazil; ^4^ School of Nutrition Federal University of Goias Goiânia Goiás Brazil

**Keywords:** autonomic function, cardiovascular system, gestational diabetes, glucose intolerance, gut microbiota, inflammation, nutraceuticals

## Abstract

This study investigated the effects of a nutraceutical combining jabuticaba peel (*Myrciaria jaboticaba* (Vell.) Berg) and *Limosilactobacillus fermentum* strains on gut microbiota, glycaemic and lipid profiles, and inflammatory markers in rats with gestational diabetes mellitus (GDM), as well as its effects on cardiac autonomic function in the offspring. Pregnant Wistar rats were assigned to control (CTL), GDM, and GDM receiving the nutraceutical (GDM+Nut). The nutraceutical (2 g/kg) or vehicle (PBS) was administered twice daily. After weaning, the offspring were fed laboratory chow until 80 days of age. Nutraceutical administration improved glucose tolerance and reduced serum inflammatory cytokines (TNF‐α and IL‐6). Beta diversity was increased without altering alpha diversity indices in the GDM+Nut. Nutraceuticals increased the abundance of short‐chain fatty acid‐producing bacteria. Maternal supplementation protected against autonomic dysfunction in the offspring. Nutraceutical supplementation shows potential as an alternative therapy for the treatment of GDM, while mitigating autonomic dysfunction in offspring.

## Graphical abstract

Maternal supplementation with a synbiotic nutraceutical consisting of jabuticaba peel and *Limosilactobacillus fermentum* strains during gestational diabetes mellitus (GDM) induces targeted shifts in gut microbial structure and attenuates systemic inflammatory signaling. In GDM dams, the intervention selectively enriches short‐chain fatty acid‐producing taxa and increases β‐diversity, aligning with improved glucose tolerance and a marked reduction in circulating TNF‐α and IL‐6. These microbiota‐dependent metabolic improvements extend to the offspring, in whom maternal synbiotic intake prevents GDM‐associated impairments in cardiac autonomic regulation. Collectively, these findings highlight the capacity of a food‐derived synbiotic to modulate host‐microbiota interactions during pregnancy, mitigating GDM‐induced metabolic stress and conferring long‐term cardiometabolic benefits to the progeny.

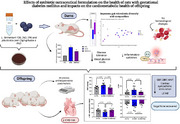

## 1 Introduction

Glycemic disorders are common during pregnancy, with approximately 16% of live births occurring to women who have had episodes of hyperglycemia during pregnancy [1]. The International Diabetes Federation estimates that 14% of pregnancies are affected by gestational diabetes mellitus (GDM), representing around 18 million cases annually [2]. The COVID‐19 pandemic caused a 38% increase in the prevalence of GDM, mainly due to stressful situations and lifestyle changes [3]. Offspring exposed to a hyperglycemic intrauterine environment are at an increased risk of developing cardiovascular and metabolic diseases in adulthood, including obesity, arterial hypertension, and type 2 diabetes mellitus [1].

Alterations in the gut microbiota (GM) during pregnancy contribute to the development of systemic disorders such as gestational glucose intolerance, insulin resistance, hormonal dysregulation, and increased levels of inflammation and oxidative stress. These disruptions can have both immediate and long‐term harmful effects on maternal and fetal health [2, 3, 4].

Studies have shown that probiotic administration can effectively reduce gut dysbiosis, plasma glucose, insulin levels, improve lipid parameters, and reduce inflammation and oxidative stress in pregnant women with GDM [5, 6]. These benefits also extend to fetal outcomes, such as a reduced incidence of fetal macrosomia and hyperbilirubinemia [7, 8]. Similarly, prebiotics consumption has been shown to modulate GM, reduce glucose absorption, and improve insulin sensitivity in GDM [9, 10]. These findings suggest that the combined intake of pro‐ and prebiotics may be a beneficial therapeutic strategy for managing GDM [11, 12, 13].

Developing nutraceuticals combining probiotics and prebiotics has been increasingly explored [14, 15, 16]. Jabuticaba is a fruit native to Brazil, recognized for its round shape, dark purple skin, and white, juicy pulp. It has a sweet taste with hints of astringency and mild acidity. Research indicates that Jabuticaba is a source of anthocyanins, tannins, phenolic acids, flavonoids, and dietary fiber with relevant hypoglycemic and anti‐inflammatory properties [17, 18]. The nutraceutical evaluated in this study comprises a combination of the probiotic strains *Limosilactobacillus fermentum* 139, *L. fermentum* 263, and *L. fermentum* 296 and jabuticaba (*Myrciaria jaboticaba* (Vell.) Berg) peel [16]. Preliminary, in vitro tests have demonstrated its potential in modulating the human GM and increasing the production of short‐chain fatty acids (SCFAs) and bioactive metabolites during colonic fermentation [16]. However, its effects on glycemic control and inflammation remain unexplored.

It has been reported that *L. fermentum* strains can modulate GM and reduce dyslipidemia, oxidative stress, and inflammation in rats fed a high‐fat diet [19, 20]. Furthermore, jabuticaba peel is rich in fiber, vitamins, minerals, and phenolic compounds (e.g., anthocyanins, polyphenols, tannins and flavonoids) that confer a range of health benefits [21]. Studies have shown that jabuticaba peel supplementation may be effective in improving lipid profile, glucose metabolism, oxidative stress, and inflammatory markers [22, 23, 24].

The use of hypoglycemic agents, such as metformin and glyburide, during pregnancy is limited due to their ability to cross the placenta [25], and the long‐term effects of intrauterine exposure to these agents on offspring are poorly understood [26]. Therefore, alternative strategies for managing GDM are essential to mitigate the risks of diseases and complications in the mother‐baby binomial. This study aimed to investigate the effects of a synbiotic nutraceutical formulation containing freeze‐dried Jabuticaba peel (*M. jaboticaba* (Vell.) Berg) and *L. fermentum* strains on the treatment of GDM in rats, as well as its impact on the cardiometabolic health of offspring. Secondary outcomes, including GM composition, lipid and glycemic profiles, and inflammatory biomarkers, were also evaluated in dams.

## 2 Materials and Methods

### 2.1 Animals and Ethical Aspects

Thirty‐six female Wistar rats (*Rattus norvegicus*), aged 60 days, were housed in polypropylene cages with filtered water and food ad libitum, on a 12‐h light‐dark cycle, with temperature (22°C ± 1°C) and controlled humidity (55% ± 10%). All experimental procedures were approved by an Ethics Committee of the Federal University of Paraíba (protocol number 2218270124) and followed the recommendations of the National Council for the Control of Animal Experimentation (CONCEA) and the International Principles for Biomedical Research Involving Animals. Efforts were made to reduce the number of animals and their suffering according to the good practice guidelines.

### 2.2 Preparation of the Synbiotic Nutraceutical

The synbiotic nutraceutical consisted of *L. fermentum* 139, *L. fermentum* 263, *L. fermentum* 296, jabuticaba peel, and fructooligosaccharide (FOS) [16]. Samples of jabuticaba cultivar Sabará (*M. jaboticaba* (Vell.) Berg, were collected, selected, sanitized with sodium hypochlorite (150 ppm, 15 min), and pulped. The peels were separated, frozen (−20°C), and freeze‐dried at −56°C for 20 h with a freeze‐drying speed of 1 mm/h (Liotop, São Paulo, SP, Brazil). After freeze‐drying, the dehydrated peels were crushed, sieved, and stored in hermetically sealed metalized bags under refrigeration (4°C ± 0.5°C) [16]. A previous study showed that Jabuticaba exerted effects on human intestinal microbiota compatible with prebiotic compounds, supporting its possible use as a functional ingredient for food and dietary supplement formulations [27].

*L. fermentum* 139, 263, and 296 strains were previously isolated from fruits, namely mango, pineapple, and strawberry, respectively [28]. Previous studies by our research group have shown that these *L. fermentum* strains are safe and have antioxidants, anti‐inflammatory, and hypolipemic properties [29, 30, 31]. The strains are deposited in the repository of probiotic strains of the Food Microbiology and Biochemistry Laboratory of the Federal University of Paraíba, Brazil (https://www.ccs.ufpb.br/lmba/).

Each strain was grown anaerobically (37°C, for 20 to 24 h) in MRS broth (AnaeroGen, Oxoid Anaerogen Anaerobic System, Basingstoke, Hampshire, UK), centrifuged for 10 min (1696 × *g*, 4°C), and washed twice with sterile saline (NaCl, 0.85%, w/v). Each strain was resuspended in sterile distilled water and mixed in equal proportions (1:1:1, v/v) to obtain a cell suspension with an optical density at 625 nm of 2.5 and a viable cell count of approximately 11 log CFU/mL.

Distilled water was added to the mixed suspension of freeze‐dried *L. fermentum* strains together with lyophilized Jabuticaba peel (17 g) and FOS (20% w/v, a well‐recognized cryoprotectant for bacterial cells) to produce the nutraceutical. The mixture was frozen at −80°C for 24 h and lyophilized for 30 h in a benchtop lyophilizer (Liotop, model L‐101; temperature −56°C ± 2°C, vacuum pressure < 170 µHg, lyophilization rate 1 mm/h) and stored under refrigeration (4°C ± 0.5°C) with light protection. The physical and chemical composition of the nutraceutical can be found in a previous study [16].

### 2.3 Experimental Design

#### 2.3.1 Mothers

**FIGURE 1:**
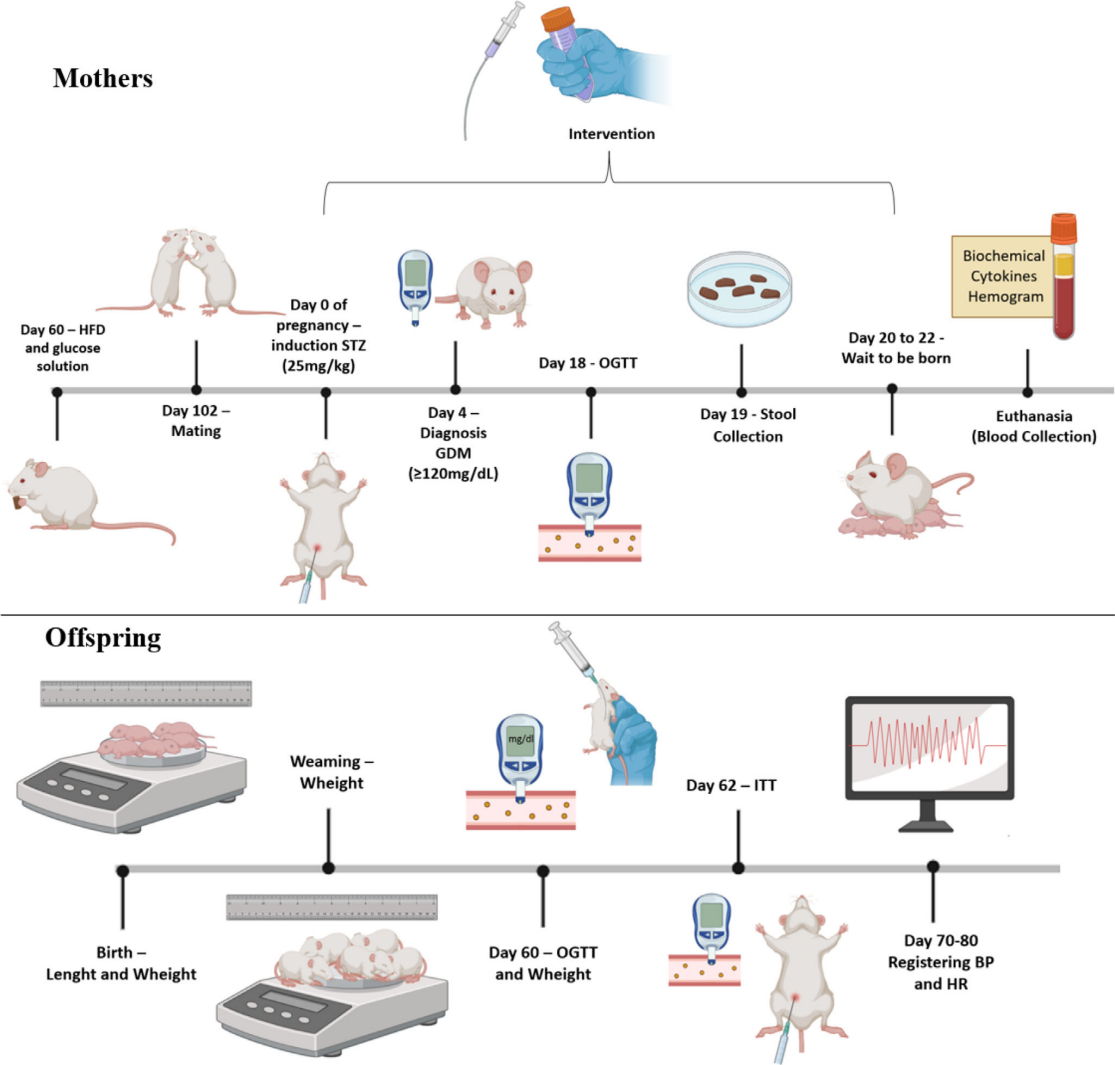
Experimental design of mothers and offspring. BP, blood pressure; HFD, high fat diet; HR, heart rate; ITT, insulin tolerance test; OGTT, oral glucose tolerance test; STZ, streptozotocin.

Female rats were randomly assigned to three groups: (i) the control group (CTL, *n* = 12), which received a control diet prepared according to the American Institute of Nutrition—AIN‐93 G; (ii) gestational diabetes + vehicle group (GDM, *n* = 12); and (iii) gestational diabetes + nutraceutical group (GDM+Nut, *n* = 12), receiving the tested nutraceutical. The GDM and GDM+Nut groups received a high‐fat diet (HFD) purchased from Rhoster (Araçoiaba da Serra, São Paulo, Brazil) and a 20% sucrose solution. Compositions of the CTL diet and HFD are shown in Table 1. The tested synbiotic nutraceutical containing *L. fermentum* strains and jabuticaba peel (2 g/kg) or vehicle (phosphate buffered solution, PBS) was administered by intragastric gavage twice daily until the end of gestation. Body weight and food consumption were measured twice weekly throughout the treatment period. Other experimental protocols included glucose tolerance tests, fecal content collection, hematological and biochemical assays, and measurement of inflammatory cytokines (Figure 1).

**TABLE 1:** Composition of control and high‐fat diet (HFD) offered.

Ingredients (g/100 g)	Diets	
	Control (AIN‐93 M)^a^	HFD^b^
Corn starch	39.75	33.09
Dextrinized corn starch	13.20	15.50
Casein^d^	20.00	19.86
Sucrose	10.00	6.00
Soybean oil	7.00	3.00
Animal fat (lard)	0.00	6.00
Non‐hydrolyzed vegetable fat	0.00	5.00
Sigma cholesterol	0.00	1.00
Sigma colic acid	0.00	0.50
Cellulose	5.00	5.00
Mineral mix 93M	3.50	3.50
Vitamin mix	1.00	1.00
L‐cystine	0.30	0.30
Choline bitartrate	0.25	0.25
t‐BHQ^c^	0.014	0.014
Nutritional composition		
Calories (Kj/100 g)	16.46	18.05
Carbohydrate (%)	63.8	50.5
Protein (%)	20.3	18.3
Lipids (%)	15.9	31.2

^a^ Adapted from Reeves et al. (1993).

^b^ Rhoster—Industry and Trade Ltd.

^c^ t‐BHQ: tert‐butylhydroquinone.

^d^ Casein showed 85% purity (85 g protein for each 100 g casein).

#### 2.3.2 Offspring

Litters were reduced to four males and four females per dam, which were named OCTL, OGDM, and OGDM+Nut groups. After weaning, offspring were fed a standard laboratory diet. Weight and length were determined from birth to the end of the experiment using an electronic laboratory balance (PFB 6000‐0 M—KERN & SOHN) and a tape measure (MedCenter). Oral glucose tolerance test (OGTT) was performed at 60 days of age, and the insulin tolerance test (ITT) after 72 h. At 70–80 days of age, vascular catheters were implanted for blood pressure and heart rate, and autonomic function assessment. After recording, rats were euthanized with an overdose of intraperitoneal pentobarbital sodium (180 mg/kg) (Figure 1).

### 2.4 Induction of GDM

After 6 weeks of HFD and 20% sucrose solution in drinking water, females were mated in a 1:1 male‐to‐female ratio. Daily vaginal smears were performed to confirm pregnancy by the appearance of sperm or a mucus plug observed by microscopy. When successful mating was established, Day 0 of gestation was considered, and pregnant rats were fasted for 10 h. After this period, female rats received an intraperitoneal injection of streptozotocin (STZ) (Sigma–Aldrich) at a single dose of 25 mg/kg to induce a model of GDM. Blood glucose was monitored before and after STZ administration (72 h later) using a blood glucose meter (Accu Chek Active, São Paulo, Brazil). Female rats with blood glucose concentrations >120 mg/dL were used as the GDM model [32]. During pregnancy, female rats were housed in separate cages until the end of the experiment. After 1‐week, non‐pregnant rats from all groups were euthanized following ethical aspects. The use of this rat model is relevant to understanding the GDM effects for both dams and offspring and identifying the effectiveness of nutraceuticals for GDM treatment before a translational approach in humans, leading to insights into the metabolic and GM changes that occur in GDM.

### 2.5 Glucose and Insulin Tolerance Tests

On gestational Day 18, an OGTT was conducted between 13:00 and 14:00 h. Rats were fasted for 6 h before OGTT, and a glucose solution with 2 g/kg body weight was administered by intragastric gavage. Blood samples were taken from the tip of the tail at 0 (baseline), 15, 30, 60, and 120 min after glucose administration using a glucose meter (ACCU‐CHEK, Roche, Germany).

ITTs were performed on 6‐h fasted offspring. They received an intraperitoneal injection of 0.75UI/kg insulin, and blood samples from the tip of the tail were taken at times 0 (baseline), 15, 30, 60, 90, and 120 min, using a glucose meter (ACCU‐CHEK, Roche, Germany) [33].

### 2.6 Hematological, Biochemical, and Cytokine Analyses in Dams

At the end of the experimental period, blood samples collected from dams were used for hematological analyses (EDTA tubes) and biochemical assays. The parameters included erythrocytes, hematocrit, hemoglobin, mean corpuscular volume (MCV), mean corpuscular hemoglobin (MCH), mean corpuscular hemoglobin concentration (MCHC), and total and differentiated leukocytes (hematology analyzer SK 9600, HoffmannLab, São Paulo, SP, Brazil). The samples were centrifuged at 3500 rpm for 15 min for the biochemical analyses. Serum levels of alanine aminotransferase (ALT), aspartate aminotransferase (AST), creatine, urea, total cholesterol, triglycerides, HDL‐cholesterol, and LDL‐cholesterol were measured. These parameters were measured with specific kits (Labtest Diagnóstica, Lagoa Santa, MG, Brazil), according to the manufacturer's recommendations, using the AMA‐B‐280 S automatic biochemical analyzer (Ama Medical, Brazil).

Cytokine levels (TNF‐α, IL‐1β, IL‐6, and IL‐10) in serum samples were determined using the Milliplex 7‐plex kit (Millipore Corp., Billerica, MA, USA). Cytokine concentrations in the samples were estimated from a standard curve using a third‐order polynomial equation and expressed in pg/mL. Samples below the detection limit of the assay were recorded as zero. In contrast, samples above the highest quantification limit of the standard curve were assigned to the highest value on the curve. Reading was performed on a CytoFLEX cytometer (São Paulo, SP, Brazil).

### 2.7 Gut Microbiota Assessment in Dams

Fecal samples were collected on Day 19 of gestation. Fecal samples were immediately placed in sterile tubes for rapid freezing and then transferred to a −80°C refrigerator for storage until DNA extraction. DNA was extracted using an exclusive magnetic bead method (Neoprospecta Microbiome Technologies, Brazil). Library preparation was performed using Neoprospecta's NGS protocol [34, 35]. Bacterial diversity was assessed by high‐throughput sequencing of the 16S rRNA V3/V4 region using primers 341F (CCTACGGGRSGCAGCAG) and 806R (GGACTACHVGGGTWTCTAAT). Libraries were quantified using the Qubit fluorometric system and by qPCR from standard curves using the Colibri Library Quantification Kit (Invitrogen, USA). The samples were sequenced using the NextSeq P1 600‐cycle kit with 2 × 305 bp paired‐end reactions. After sequencing, the sequences were analyzed using a proprietary pipeline (Neoprospecta Microbiome Technologies, Brazil). Briefly, the sequences were demultiplexed and subjected to a quality filter based on the sum of the error probabilities of the DNA bases, allowing a maximum of 1% accumulated errors. The Illumina adapters and primers were removed from the DNA sequences. All 16s rRNA Illumina amplicon sequencing data provided in this study can be publicly obtained from the Sequence Read Archive (SRA) of the National Center for Biotechnology Information (NCBI) under the accession number PRJNA1200614.

### 2.8 Determination of Baseline Cardiovascular and Assessment of Sympathetic Tone in Offspring

Rats were anesthetized intraperitoneally with ketamine (75 mg/kg) and xylazine (10 mg/kg) to implant polyethylene catheters into the femoral artery and femoral vein. The catheters were tunneled into the dorsal scapular region of the animal, and ketoprofen (5 mg/kg) was injected subcutaneously. The rats underwent a surgical recovery period of 24 h, after which BP and HR were recorded in conscious animals by connecting the arterial cannula to a pressure transducer (ML866/P, AD Instruments, Power Lab, Bella Vista, NSW, Australia) as previously described [36]. Pulsatile arterial pressure (PAP) was recorded for 40 min under baseline conditions, and mean arterial pressure (MAP), systolic arterial pressure (SBP), diastolic arterial pressure (DBP), and HR values were selected from 10 min segments of the recording for each animal (LabChart TM Pro, ADInstruments, Bella Vista, NSW, Australia).

Autonomic function was assessed by administration of atropine (2 mg/kg), a muscarinic antagonist that acts as a modulator of parasympathetic function, and a β‐blocker called propranolol (10 mg/kg) to assess sympathetic autonomic function. Selected sections of the baseline BP and HR recordings were used for frequency domain spectral analyses of SAP and Pulse Interval (PI). The SAP spectra were integrated into the LF (0.2–0.75 Hz) and HF (0.75–3 Hz) bands. The LF/HF ratio of the PI was used to assess the sympathovagal index. Analyses were performed using CardioSeries software (v.2.4; www.danielpenteado.com) [19].

### 2.9 Statistical Analysis

Data were analyzed using GraphPad Prism 8.0 (GraphPad Software, San Diego, CA, USA) for most data analysis and R software (version 4.4.2) for GM analysis. The Kolmogorov–Smirnov test was used to analyze the data normality. Results were expressed as mean ± standard deviation for parametric tests. One‐way ANOVA and Tukey's post‐test were used to compare the groups. Non‐parametric results were expressed as median (maximum and minimum), and Kruskal–Wallis with Dunn's post‐test was used to compare groups. Two‐way ANOVA with Bonferroni post‐test was used to compare body weight during pregnancy and OGTT. The difference was considered significant when *p* < 0.05. Spearman's correlation was used to analyze GM diversity and biochemical and inflammatory data. The correlations were classified as bad (r ≤ 0.20), weak (0.21–0.40), moderate (0.41–0.60), good (0.61–0.80), and excellent (0.81–1.00).

Sequences from the GM analyses were “demultiplexed” using Bcl2fastq software (v2.17.1.14) and then cleaned using cutadapt (v1.9.1). Amplicon sequence variants (ASVs) were identified using Qiime2 DADA2 with a zero‐noise OTU approach. Finally, the taxonomic assignment was performed using the Greengenes 13.8‐nr99 base. After sequencing the GM samples, the sequences were imported into R software for statistical analyses, using specific packages. Alpha and beta diversities were analyzed, with beta diversity analysis performed using the Unifrac and Bray‐Curtis methods. Phyla, family, and genus analysis were performed using Maaslin2 package and Kruskal–Wallis statistical tests with Dunn's post‐test, and *p* < 0.05 was considered significant.

## 3 Results

### 3.1 Synbiotic Nutraceutical Formulation Did Not Alter Food Consumption or Gestational Weight Gain

**FIGURE 2:**
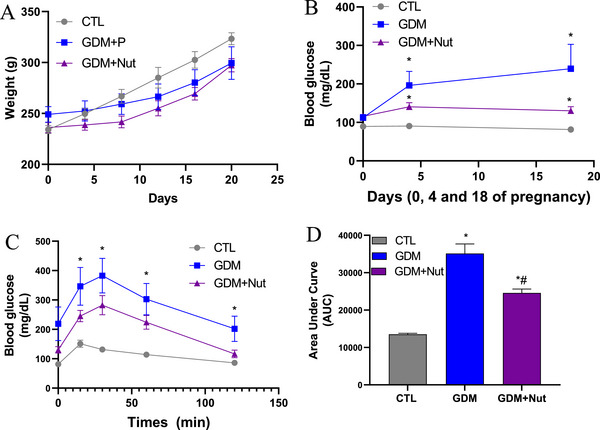
Gestational weight gain, glycaemia and glucose tolerance in healthy pregnant rats (CTL), rats with gestational diabetes mellitus (GDM), and rats with GDM treated with nutraceutical during pregnancy twice a day (GDM+Nut). (A) Body weight during pregnancy. (B) Glycemia on Days 0, 4, and 18 of pregnancy. (C) Oral glucose tolerance test. (D) The area under the curve (AUC) was calculated as a glucose tolerance index. Data are expressed as mean ± standard deviation and analyzed by two‐way ANOVA with Bonferroni as a post hoc test or one‐way ANOVA with Tukey as a post hoc test. *p < 0.05 vs. O‐CTL; #*p* < 0.05 vs. O‐GDM.

At the end of the experiments, the CTL group was formed with *n* = 9 dams, the GDM group was formed with *n* = 8 dams, and the GDM+Nut group was formed with *n* = 8 dams. Food consumption throughout the experiment was not significantly different between groups (CTL: 25.39 ± 4.81 g; GDM: 16.79 ± 2.77 g; GDM+Nut: 16.29 ± 0.15 g, *p* > 0.05). Similarly, no significant differences were observed in gestational weight gain among the groups (CTL: 276.9 ± 33.31 g; GDM: 267.8 ± 19.09 g; GDM+Nut: 256.4 ± 23.57 g, *p* = 0.41, F = 0.94, Figure 2A).

### 3.2 Synbiotic Nutraceutical Improved Glucose Tolerance in Dams

Blood glucose levels on Days 0, 4, and 18 of gestation were higher in the GDM group compared to the CTL group (89.63 [81.38–90.38] vs. 187.6 [116.7–218.9] mg/ dL, *p* = 0.003, Figure 2B). The treatment with synbiotic nutraceutical reduced blood glucose levels (130 [115.9–140.6], *p* < 0.05, Figure 2B) when compared to the GDM group. In addition, the nutraceutical treatment reduced OGTT in GDM dams (*p* = 0.0002, Figure 2C,D).

### 3.3 Synbiotic Nutraceutical Formulation Did Not Affect Hematological and Lipid Profile Parameters But Significantly Reduced Serum Inflammatory Cytokines in Dams

Leukocytes and segmented leukocytes showed a significant increase in the GDM group compared to the CTL group, while no differences were observed between the GDM+Nut group and the CTL group (Table 2). Erythrocytes, hematocrit, hemoglobin, and lymphocytes did not differ among groups (Table 2). The GDM group had elevated total cholesterol, LDL‐cholesterol, triglycerides, serum creatinine, AST, and ALT compared to the CTL group (Table 2). No differences in these markers were observed between the GDM+Nut and CTL groups (Table 2). Inflammatory cytokine analysis revealed a significant reduction in TNF‐α and IL‐6 levels in the GDM+Nut compared to the GDM group (*p* < 0.05, Table 2), whereas IL‐10 was significantly lower in the GDM group than in the CTL group (*p* < 0.05, Table 2).

**TABLE 2:** Hematological, biochemical and inflammatory parameters of healthy pregnant rats (CTL), with GDM receiving vehicle (GDM), and with GDM treated with nutraceutical (GDM+Nut—Lyophilized *L. fermentum* 139, 263, 296 and jabuticaba peel) twice a day during pregnancy.

Hematological parameters	CTL	GDM	GDM+Nut	*p* value
Erythrocytes (10^6^ / mm^3^)	5.4 (5.2–5.8)	5.2 (5.1–5.5)	5.3 (5.2–5.5)	0.26
Hematocrit (%)	41.0 (40.8–43.0)	42.0 (41.3–42.5)	42.0 (40.8–42.7)	0.73
Hemoglobin (%)	13.8 (13.0–14.0)	12.9 (12.2–13.5)	13.7 (13.0–14.0)	0.03
MCV (%)	41.2 (40.6–42.7)	42.5 (41.5–44.0)	40.8 (39.7–42.0)^#^	0.01
MCH (%)	15.3 (14.9–15.6)	15.4 (14.8–16.0)	15.8 (15.0–16.4)	0.53
MCHC (%)	35.2 (34.9–35.6)	35.0 (34.7–35.3)	34.2 (33.5–35.2)	0.2
Leukocytes (10^3^ / mm^3^)	8.0 (7.9–8.2)	8.3 (8.2–8.5)^*^	8.3 (8.2–8.4)	0.01
Segmented (%)	53.4 (52.4–54.1)	57.9 (55.5–59.3)^*^	56.3 (54.2–58.9)	0.001
Lymphocytes (%)	33.5 (32.6–34.6)	34.5 (33.7–35.0)	32.3 (30.7–34.8)	0.09
Monocytes (%)	9.4 (9.2–9.8)	8.64 (8.30–8.8)^*^	8.3 (8.0–8.8)^*^	0.0005
**Biochemical parameters**				
Triglycerides (mg/dL)	103.3 (98.3–107.3)	195.4 (187.0–205.3)^*^	134.6 (128.4–142.4)	<0.0001
Total cholesterol (mg/dL)	109.8 (102.4–117.7)	219.4 (197.9–254.7)^*^	188.6 (165.5–199.7)	<0.0001
LDL‐cholesterol (mg/dL)	21.9 (20.4–23.5)	92.1 (83.1–102.4)^*^	56.5 (49.6–58.3)	<0.0001
HDL‐cholesterol (mg/dL)	49.4 (46.0–52.9)	68.0 (61.3–78.9)	84.8 (79.1–89.8)^*^	<0.0001
Creatinine (mg/dL)	0.7 (0.6–0.8)	0.9 (0.8–1.1)^*^	0.7 (0.6–0.8)	0.008
Ureia (mg/dL)	31.1 (27.7–34.4)	35.4 (33.2–39.0)	33.2 (30.1–35.0)	0.08
AST (U/L)	44.5 (42.2–48.1)	70.3 (67.8–95.3)^*^	53.6 (51.5–58.3)	<0.0001
ALT (U/L)	44.1 (40.8–49.5)	72.3 (61.8–90.3)^*^	49.6 (45.3–52.3)	<0.0001
**Cytokines levels**				
TNF‐α (pg/mL)	72.2 (69.7–76.5)	91.2 (86.4–97.1)	70.2 (68.3–74.7)^#^	0.0006
IL‐1β (pg/mL)	69.4 (67.4–74.9)	71.9 (68.6–77.3)	72.1 (65.2–75.4)	0.48
IL‐6 (pg/mL)	51.5 (48.7–53.2)	65.1 (59.2–78.5)	46.2 (44.2–53.8)^#^	0.0003
IL‐10 (pg/mL)	70.8 (68.0–74.2)	50.0 (48.7–53.3)^*^	65.2 (62.3–70.4)	<0.0001

*Note*: Data are expressed as median (minimum–maximum) and analyzed by the Kruskal–Wallis test with Dunn as a post hoc test.

Abbreviations: ALT, alanine aminotransferase; AST, aspartate aminotransferase; HDL, high density lipoprotein; IL, interleukin; LDL, low density lipoprotein; MCH, mean corpuscular hemoglobin; MCHC, mean corpuscular hemoglobin concentration; MCV, mean corpuscular volume; TNF‐α, tumor necrosis factor‐alpha.

^*^ *p* < 0.05 vs. CTL group; ^#^ *p* < 0.05 vs. GDM group.

### 3.4 Synbiotic Nutraceutical Improves Gut Microbiota Diversity and Composition in Dams

**FIGURE 3:**
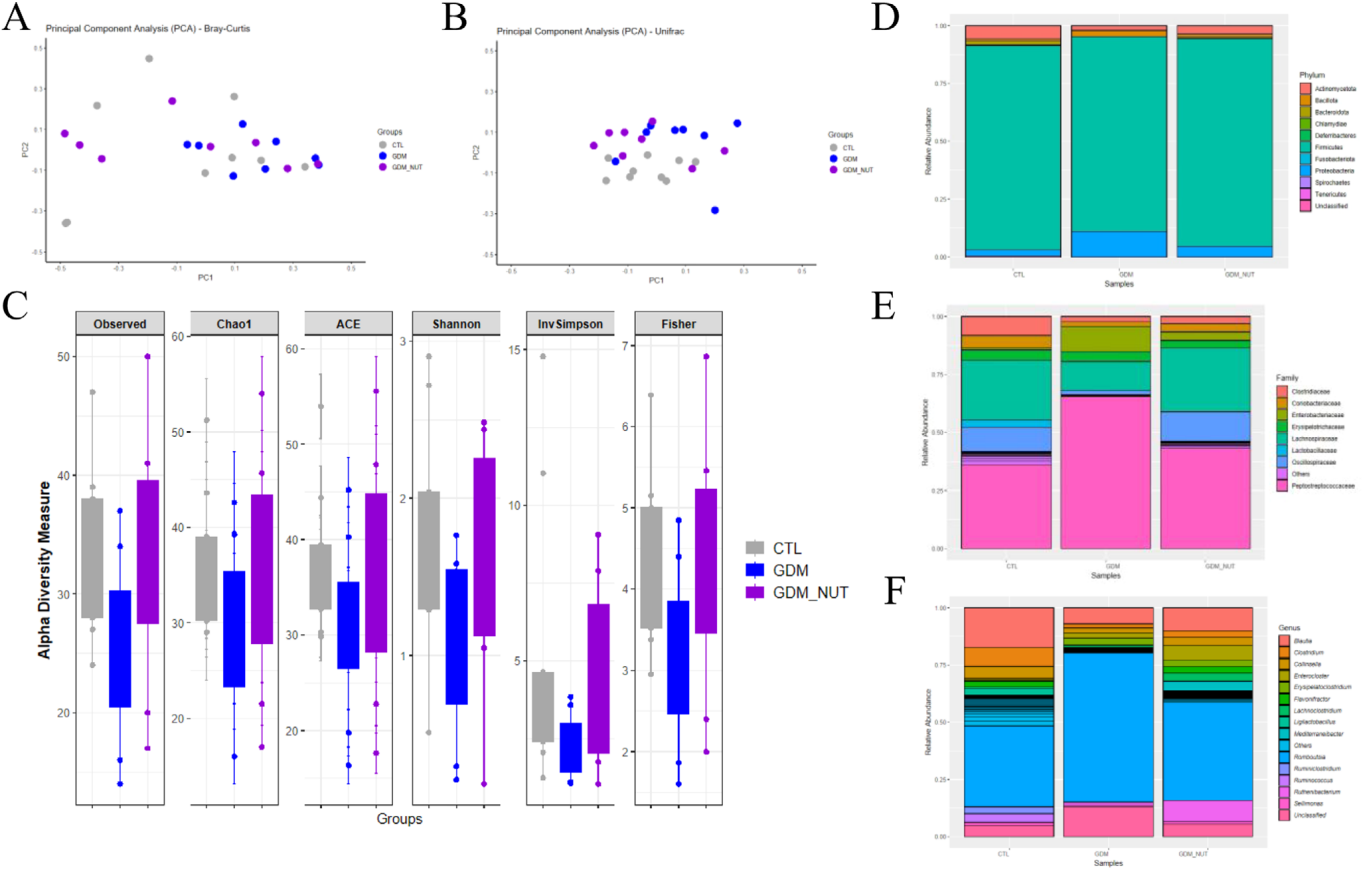
Administration of the nutraceutical containing freeze‐dried jabuticaba peel and *L. fermentum* induces changes in the diversity and composition of the gut microbiota in GDM rats. (A) and (B) CoA analysis based on Bray‐Curtis and Unifrac dissimilarity was used to represent the beta diversity of each group. (C) Alpha diversity was determined using the Chao1 (Richness), ACE (Richness), Shannon (richness and evenness), Simpson and Fisher (number of species and the evenness). Relative abundance of bacterial microbiota composition at the (D) phylum, (E) families, and (F) genus. Significance between groups was calculated using the non‐parametric Kruskal–Wallis and Maaslin tests.

**FIGURE 4:**
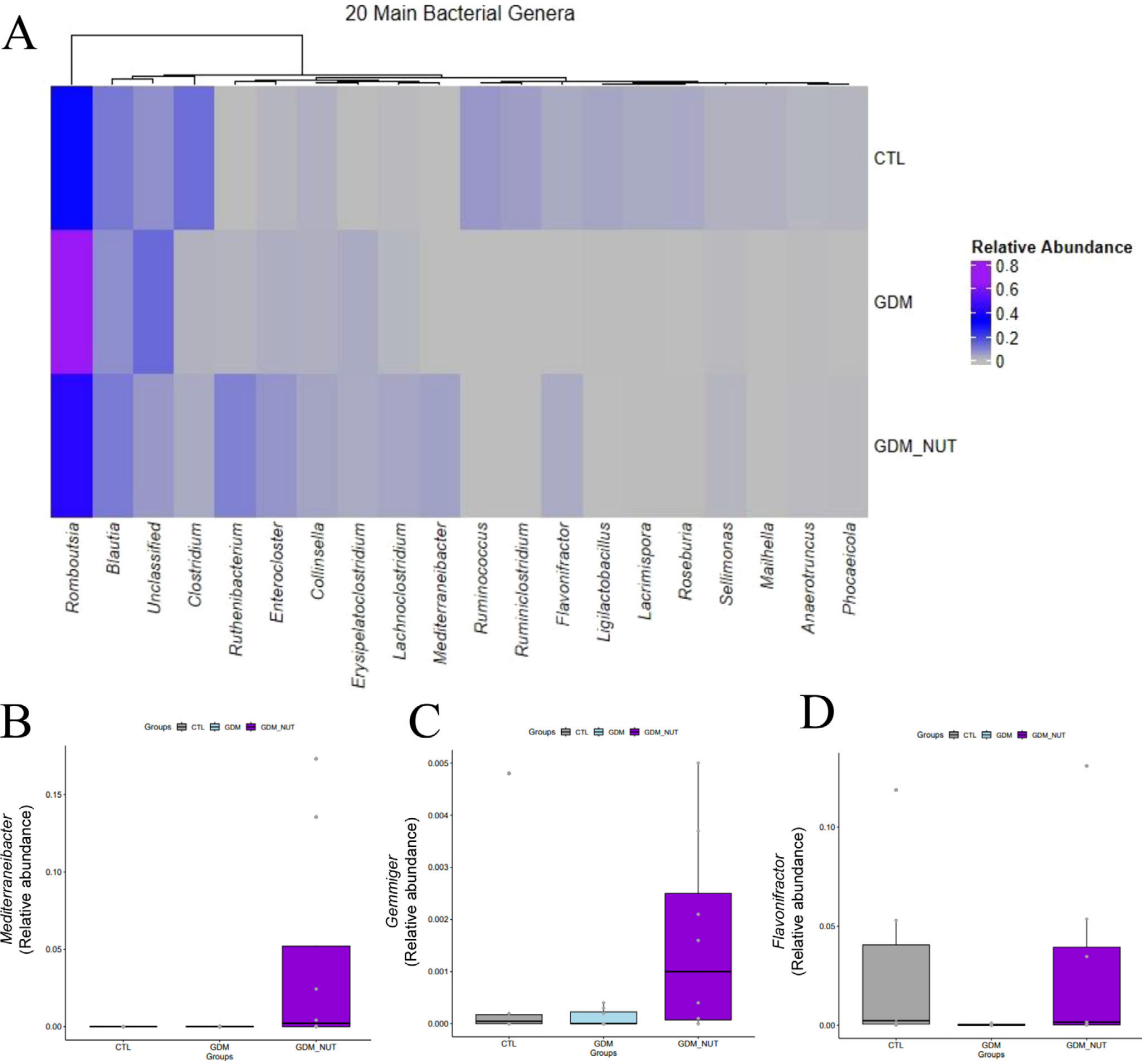
Effects of a mixed formulation freeze‐dried Jabuticaba peel and *L. fermentum* on gut microbiota. Heatmap showing the relative abundance of microbial taxa at the genus level. Groups: control group (CTL), gestational diabetes mellitus receiving vehicle (GDM), and GDM receiving formulation freeze‐dried Jabuticaba peel and *L. fermentum* (GDM+Nut) twice a day during gestation.

Analysis of GM clustering revealed significant differences in Bray‐Curtis dissimilarity (F = 5.98, *p *= 0.009), but not in UniFrac distances (F = 1.31, *p *= 0.29) (Figure 3A,B). The GDM group had lower richness and alpha diversity, as assessed by the Chao, Shannon, Simpson, and Fisher indexes, compared to the CTL group (*p* < 0.05, Figure 3C). The nutraceutical treatment did not increase alpha diversity indexes (Chao: *p *= 0.11; Shannon: *p *= 0.09; Fisher: *p *= 0.11). Relative abundance analysis identified 11 bacterial phyla, with Firmicutes, Actinomycetota, Proteobacteria, Bacteroidota, Bacillota, Fusobacteriota, and Tenericutes being the most abundant (Figure 3D). GDM rats showed a decrease in the relative abundance of Tenericutes and Bacteroidota (*p* < 0.05, Figure 3D). Treatment with the nutraceutical formulation increased Bacteroidota abundance compared to the GDM group (*p* < 0.05, Figure 3D). At the family level, Clostridiaceae, Coriobacteriaceae, Enterobacteriaceae, Erysipelotrichaceae, Lachnospiraceae, Lactobacillaceae, Oscillospiraceae were the most abundant (Figure 3E). The GDM group showed a decrease in the relative abundance of Lactobacillaceae, Helicobacteriaceae, Corynebacteriaceae, Mycoplasmataceae, alongside an increased abundance of Pectobateriaceae, Peptostreptococcaceae compared to the CTL group (*p* < 0.05). The nutraceutical formulation decreased the relative abundance of Staphylococcaceae and increased Coprobacillaceae compared to the GDM group (*p* < 0.05). The most abundant genera identified are presented in Figure 3F, while a heatmap illustrating the top 20 genera is shown in Figure 4A. The GDM group showed a reduction in the relative abundance of the genus *Mycoplasma*, *Helicobacter*, *Anaerotignum*, *Faecalibaculum*, *Flintibacter*, *Ruminoclostridium*, Phocaeicola, *Clostridium*, *Butyricimonas*, *Bacteroides*, *Flavonifractor*, *Lactobacillus*, *Fusicatenibacter*, *Bifidobacterium*, *Roseburia*, and *Ruminococcus* compared to the CTL group (*p* < 0.05). Conversely, the GDM group showed an increased relative abundance of the genera *Erysipeloclostridium* and *Romboutsia* compared to the CTL group (*p* < 0.05). Treatment with synbiotic nutraceutical increased the relative abundance of *Mediterraneibacter*, *Anaerostipes*, *Holdemania*, *Dysosmobacter*, *Flavonifractor*, Phocaeicola, and *Gemmiger* compared to the GDM group (*p* < 0.05, Figure 4B–D).

Spearman correlation test (Table 3) shows significant negative associations between alpha diversity indices and biochemical and inflammatory variables in dams. A weak negative correlation was found between the AUC‐OGTT and the Shannon index. A moderate negative correlation trend was observed between Chao1, ACE, Shannon, and Fisher indices and serum ALT, AST, and IL‐6 levels. Serum creatinine levels were negatively correlated with all alpha diversity indices. Serum IL‐10 was positively correlated with Shannon's, Simpson's, and Fisher's indices. When evaluating the lipid profile, a negative correlation was observed between triglycerides and LDL‐c and the Fisher index and LDL‐c and the Shannon index.

**TABLE 3:** Correlation of alpha diversity indices and biochemical and inflammatory parameters in dams.

	Chao1	ACE	Shannon	Simpson	Fisher
AUC‐OGTT	−0.21	−0.20	−0.40*	−0.38	−0.26
ALT	−0.43*	−0.41*	−0.39*	−0.36	−0.46*
AST	−0.41*	−0.39*	−0.43*	−0.41*	−0.44*
Total cholesterol	−0.31	−0.31	−0.36	−0.30	−0.33
Tryglicerides	−0.33	−0.32	−0.36	−0.31	−0.37*
Creatinine	−0.54*	−0.52*	−0.52*	−0.49*	−0.56*
Ureia	−0.10	−0.09	−0.16	−0.16	−0.14
HDL‐c	−0.11	−0.13	−0.16	−0.13	−0.10
LDL‐c	−0.33	−0.33	−0.37*	−0.32	−0.37*
TNF‐α	−0.29	−0.29	−0.32	−0.26	−0.36
IL‐1B	−0.29	−0.29	−0.00	−0.05	−0.25
IL‐6	−0.44*	−0.42*	−0.42*	−0.42*	−0.47*
Il‐10	0.35	0.34	0.41*	0.37*	0.40*

*Note*: Data are expressed Spearman's correlation coefficient (*ρ*) values analyzed by the Spearman's correlation.

Abbreviations: ALT, alanine aminotransferase; AST, aspartate aminotransferase; AUC‐OGTT, area under curve‐oral glucose tolerance test; HDL, high density lipoprotein‐cholesterol; IL, interleukin; LDL, low density lipo‐protein‐cholesterol; TNF‐α, tumor necrosis factor‐alpha.

^*^ *p* < 0.05.

### 3.5 Synbiotic Nutraceutical Improved Anthropometric Parameters but Limited Metabolic Improvements in Offspring

The birth weight of the O‐GDM group was significantly lower compared to the O‐CTL group (Table 4). In contrast, the birth weight and length in the O‐GDM+Nut group were significantly higher compared to both the O‐GDM and O‐CTL groups (*p* < 0.05, Table 4). During the weaning period, male and female offspring in the O‐GDM+Nut group showed higher body weight compared to those in the O‐CTL group. By 60 days of age, female offspring in the O‐GDM+Nut group showed a significant increase in body weight compared to females in both the O‐GDM and O‐CTL groups (Table 4).

**TABLE 4:** Weight and length of the offspring of the CTL (O‐CTL), GDM (O‐GDM) and GDM+Nut (O‐GDM+Nut) groups from birth to adolescence.

	O‐CTL	O‐GDM	O‐GDM+Nut	*p* value
**Birth**				
Weight (g)	6.28 ± 0.45	5.60 ± 0.72^*^	6.90 ± 0.17^*#^	<0.0001
Lenght (cm)	4.37 ± 0.10	4.34 ± 0.33	4.74 ± 0.14^*#^	<0.0001
**Weight (g)—21 days**				
Male	35.5 ± 8.45	35.77 ± 7.01	40.64 ± 3.40	0.0556
Female	32.64 ± 6.22	35.66 ± 0.81	40.35 ± 2.06^*^	<0.0001
**Weight (g)—60 days**				
Male	215.37 ± 13.02	215.66 ± 36.98	235.87 ± 30.05	0.2818
Female	154.5 ± 10.96	164.5 ± 9.35	186.25 ± 19.94^*#^	0.0012

*Note*: Data are expressed as mean ± standard deviation and analyzed by one‐way ANOVA with Tukey as a post hoc test.

^*^ *p* < 0.05 vs. O‐CTL; ^#^ *p* < 0.05 vs. O‐GDM.

**FIGURE 5:**
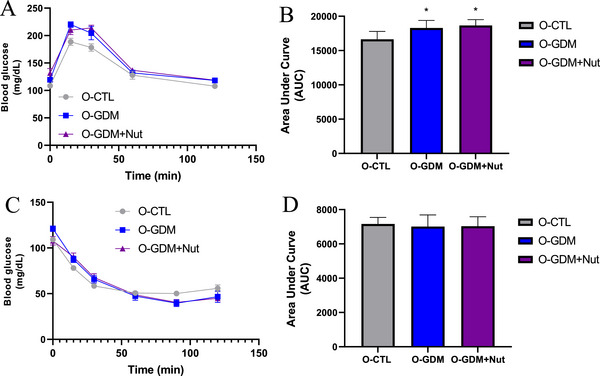
Glucose and insulin tolerance of the offspring of healthy rats (O‐CTL), rats with GDM (O‐GDM), and rats with GDM treated with a nutraceutical formulation of *L. fermentum* and jabuticaba peel (GDM+Nut). (A) Oral glucose tolerance test. (C) Insulin tolerance test. (B) and (D) The area under the curve (AUC) was calculated as an index of glucose tolerance or insulin tolerance for each test. Data are expressed as mean ± standard deviation and analyzed by one‐way ANOVA test with Tukey as post hoc test. *p < 0.05 vs. O‐CTL.

The GTT results for the offspring of control dams, GDM dams, and GDM dams treated with the nutraceutical formulation are presented in Figure 5A. Analysis of the AUC showed a significant increase in O‐GDM and O‐GDM+Nut groups compared to the O‐CTL group (*p* < 0.0001, F = 17.04, Figure 5B). However, no significant glycemic variations were observed among the groups in the ITT (*p *> 0.05, Figure 5D).

### 3.6 Improved Cardiovascular Health in Offspring of GDM Dams Treated With Synbiotic Nutraceutical

**FIGURE 6:**
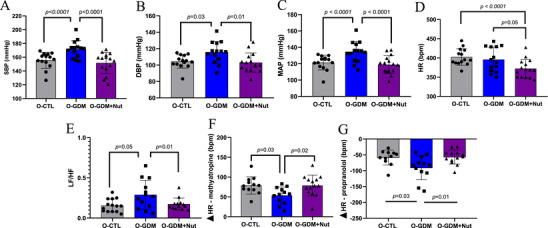
Cardiovascular health parameters of the offspring of healthy rats (O‐CTL), rats with GDM (O‐GDM), and rats with GDM treated with a nutraceutical formulation of *L. fermentum* and jabuticaba peel (GDM+Nut). The distributed dots on the graph represent the animals. (A) SBP—systolic blood pressure. (B) DBP—diastolic blood pressure. (C) MAP—mean arterial pressure. (D) HR—heart rate. (E) Low‐frequency/high‐frequency ratio. (F) Evaluation of parasympathetic function. (G) Evaluation of sympathetic function. Data are expressed as mean ± standard deviation and analyzed by one‐way ANOVA test with Tukey as a post hoc test.

Male and female offspring in the O‐GDM group had an increase in SBP (155.6 ± 11.6 vs.171.7 ± 12.4 mmHg, *p* < 0.0001, Figure 6A), DBP (104.4 ± 8.1 vs. 115.6 ± 13.1 mmHg, *p* = 0.03, Figure 6B), and MAP (121.3 ± 8.9 vs.134.3 ± 12.6 mmHg, *p* < 0.0001, Figure 6C) compared to the O‐CTL group. The administration of nutraceuticals in GDM dams (O‐GDM+Nut) significantly reduced SBP (151.6 ± 15.4 mmHg, *p* < 0.0001, Figure 6A), DBP (103.1 ± 11.7 mmHg, *p* = 0.01, Figure 6B), and MAP (118.8 ± 11.0 mmHg, *p* < 0.0001, Figure 6C) compared to the O‐GDM group. Maternal treatment with the synbiotic formulation also reduced HR in the O‐GDM+Nut (372.3 ± 23.68 bpm) compared to both the O‐CTL (402.3 ± 21.67 bpm, *p* < 0.0001) and O‐GDM (395.8 ± 32.01 bpm, *p* = 0.05) groups (Figure 6D).

Spectral analysis revealed that the O‐GDM group had a significant increase in LF/HF ratio compared to the O‐CTL (0.28 ± 0.18 vs. 0.15 ± 0.08, *p* = 0.05, Figure 6E). On the other hand, the O‐GDM+Nut group showed a significant reduction in LF/HF ratio compared to the O‐GDM group (0.17 ± 0.07, *p* = 0.01, Figure 6E).

Figure 6F,G illustrated changes in HR in response to the administration of methylatropine (vagal tone) and propranolol (sympathetic stimulus). Vagal tone was significantly decreased in the O‐GDM group compared to the O‐CTL group (78.9 ± 21.8 vs. 53.7 ± 21.2 bpm, *p* = 0.03, Figure 6F). However, vagal tone in the O‐GDM+Nut group was significantly higher compared to the O‐GDM group (78.8 ± 26.0 bpm, *p* = 0.02, Figure 6F). For sympathetic stimulation, a significant increase was observed in the O‐GDM compared to the O‐CTL group (−90.5 ± 37.8 vs. −58.4 ± 23.5, *p* = 0.03, Figure 6G). The O‐GDM+Nut group showed a significant reduction in cardiac sympathetic stimulation compared to the O‐GDM group (56.6 ± 22.4, *p* = 0.01, Figure 6G).

## 4 Discussion

This study showed that administering a synbiotic nutraceutical containing *L. fermentum* strains and jabuticaba peel twice daily during pregnancy promoted improvement in glucose tolerance, reduced inflammation, and modulated the GM in dams with GDM. Furthermore, maternal nutraceutical intervention protects offspring against increased blood pressure and autonomic dysfunction later in life.

In a previous study using an animal model of GDM induced by streptozotocin and a high‐fat and high‐sugar diet, the oral administration of galactooligosaccharide at low and high doses (0.9 and 1.8 g/kg, respectively, for 30 days) did not alter gestational weight gain [37], like the results found in this study. The consumption of probiotics and prebiotics, alone or together, has been studied in recent years regarding their physiological functions in health and disease, with glycemic control in GDM being one of the many benefits reported in the literature [11, 38, 39].

Although this study showed that administering the synbiotic nutraceutical containing *L. fermentum* and jabuticaba peel could not reduce fasting glycemia, an improvement in glucose tolerance was observed. The reduction in glucose tolerance in pregnant women with GDM is associated with an improvement in glucose metabolism, which implies a decrease in glycemic peaks and the risk of developing chronic non‐communicable diseases in their offspring [40]. A study showed that the supplementation of *Lactobacillus rhamnosus* LCG (5 × 10^8^ CFU/10 g) and *Bifidobacterium animalis* subsp. *lactis* Bb12 (1 × 10^9^ CFU/10 g) at a low and high dose (0.5 g/day and 1 g/day, for 15 days) reduced significantly fasting glycemia in rats with GDM [41]. Additionally, preclinical studies have also shown that jabuticaba may exert health benefits on insulin sensitivity, glucose and lipid metabolism, and decrease inflammation [42, 43]. Anthocyanins, essential components of fruits, provide protection and exhibit anti‐diabetic, anti‐obesity, and anti‐inflammatory effects [44].

Changes in blood cells, including monocytes, have been observed in diabetes mellitus [45, 46]. Monocytes may play a key role related to chronic inflammation in disease. Patients with type 2 diabetes mellitus have reduced levels of type 2 monocytes [46]. The hematological results of this study suggest that the tested nutraceutical may be safe for human consumption.

Previous studies have reported anti‐inflammatory effects of probiotic consumption and jabuticaba (*M. jaboticaba*) [47, 48]. Anthocyanins from jabuticaba protect against inflammation and interact with the GM [49]. Because of low absorption, polyphenols like anthocyanins reach the intestine, where the GM metabolizes them [50]. Proposed mechanisms by which phenolic compounds reduce the expression of pro‐inflammatory cytokines involve inhibiting pro‐inflammatory NF‐κB pathways and decreasing the expression of adhesion molecules [49, 51]. In addition, the increased abundance of bacterial genera and families that produce SCFA may exert an anti‐inflammatory effect [52]. SCFA binds to G protein‐coupled receptors (GPCRs) and activates histone deacetylase (HDAC), inhibiting NF‐κB signaling and pro‐inflammatory cytokines [53].

Changes in the composition or diversity of the GM can lead to beneficial metabolic changes, enhancing nutrient metabolism, insulin sensitivity, and hormone production [54]. In this study, treatment with the nutraceutical in female rats with GDM successfully increased the diversity and modified the composition of GM by boosting the relative abundance from the genera *Mediterraneibacter*, *Anaerostipes*, *Flavonifractor*, *Holdemania*, *Dysosmobacter*, *Phocaeicola*, and *Gemmiger*, while reducing the abundance of *Romboutsia*.

*Mediterraneibacter*, *Anaerostipes*, *Flavonifractor*, and *Dysosmobacter* are associated with increased production of SCFA in the gut, particularly butyrate [55, 56, 57]. A previous study indicated that species of the genus *Dysosmobacter* were more abundant in patients taking metformin and showed a negative correlation with fasting glucose levels in subjects with obesity and type 2 diabetes mellitus [58]. In addition, it was demonstrated that *Dysosmobacter welbiones* J115 supplementation (1 × 10^9^ CFU/0.2 mL for 6 weeks) was negatively correlated with BMI, HbA1c, and fasting glucose in mice fed an HFD [59].

Administration of *Phocaeicola vulgatus*, recently renamed as *Bacteroides vulgatus* (1 × 10^9^ CFU/100 µL) for 6 weeks, reduced triglycerides and hepatic steatosis in C57BL/6J mice through the production of metabolites, such as 3‐HPAA, which plays a protective role by reducing fatty acid metabolism by liver cells and inhibiting hepatic steatosis [60]. In addition, bacteria of the genus *Phocaeicola* are among the main colonizing microorganisms in the infant's intestine during vertical transmission [61]. *Phocaeicola faecalis* FXJYN30E22 showed beneficial effects in the treatment of ulcerative colitis, such as anti‐inflammatory effects, with the increase in the concentration of junctional proteins such as ZO‐1, claudin‐1, and occludin, and SCFA such as acetate and butyrate [62].

Japanese pregnant women with GDM exhibited a higher relative abundance of *Romboutsia* than those without GDM [63]. Otherwise, anti‐diabetic medications, such as Metformin and Liraglutide, decreased the relative abundance of *Romboutsia* in patients with type 2 diabetes mellitus [64, 65]. Here we have shown that female rats with GDM had a higher relative abundance of *Romboutsia*, and the nutraceutical administration decreased the relative abundance of *Romboutsia* to a similar extent as the control group. Taken together, the results of this study suggest that administering a nutraceutical containing *L. fermentum* and jabuticaba peel improves glycemic control in rats with GDM by modulating the diversity and composition of the GM.

The GM is closely linked to the immune system [66]. The development of autoimmune diseases has been associated with gut dysbiosis and decreased abundance from the genera *Gemmiger*, *Anaerostipes*, *Ruminococcus*, *Roseburia*, *Alistipes*, and *Faecalibacterium* [67]. These bacterial genera have been linked to butyrate production and anti‐aging and anti‐inflammatory effects [68]. The abundance of *Gemmiger* was diminished in patients with type 2 diabetes mellitus and diabetic nephropathy, showing a negative correlation with HbA1c and fasting glycemia [69]. The importance of butyrate in GDM is highlighted by the capacity of intestinal L‐cells to utilize SCFA as cellular energy. It is also associated with the production of GLP‐1, an insulinotropic hormone relevant for glucose metabolism [38].

In this study, we demonstrated the intimate relationship between GM alpha diversity indices and biochemical and inflammatory parameters. We found that inadequate glycemic control may be negatively associated with the Shannon index. In addition, the increase in serum levels of biochemical parameters such as ALT, AST, and creatinine in GDM rats may be negatively associated with alpha diversity indices. The association between elevated inflammatory cytokines, especially IL‐6, with the onset of GDM and intestinal dysbiosis has been shown in previous studies [3, 70]. In this study, we observed that the increase in serum IL‐6 is closely related to the diversity of the composition of the GM through a decrease in Chao1, ACE, Shannon, Simpson, and Fisher index.

The hyperglycemic intrauterine environment can influence pro‐apoptotic phenomena associated with oxidative stress and inflammation, leading to malformations in the cardiovascular, renal, and central nervous systems [71]. Impaired GM composition can alter placental structure and function, particularly nutrient transport [72, 73]. In addition, GM‐derived lipopolysaccharide (LPS) particles can cross the placenta via toll‐like receptors (TLR) 2 and TLR4, reach fetal tissues, activate inflammatory pathways leading to alterations in fetal development [74], and increase the risk of developing cardiovascular disease, such as arterial hypertension later in life [75].

Our results support previous studies showing that GDM is a risk factor for arterial hypertension in the offspring later in life due to endothelial dysfunction, and sympathetic hyperactivity [76, 77, 78]. GDM may decrease the concentration and bioavailability of nitric oxide to interact with the vascular endothelium of the offspring, impairing endothelium‐dependent vasodilation and increasing vasoconstriction. Such changes are characterized as endothelial dysfunction [79].

Previous studies have shown that maternal therapy with probiotics and polyphenols can protect against the development of hypertension in offspring [80, 81]. Although consistent beneficial effects have been reported with the administration of probiotics, polyphenols, and fiber in GDM conditions [11, 82], their impacts on the offspring of dams with GDM are poorly understood. Here, we showed for the first time that administering a nutraceutical containing probiotics and jabuticaba prevents arterial hypertension and cardiac autonomic dysfunction in male and female offspring later in life. While these findings are promising, the study's preclinical nature necessitates caution when extrapolating to human populations. The lack of direct mechanistic insight investigating whether synbiotic nutraceutical alters the glucose transport expression in the gut or glucose metabolism in pancreas, muscle, and liver in GDM condition represents a potential limitation of the study. In addition, the lack of SCFA quantification, gut hormones, and placental transfer are potential limitations of the study. In this way, future molecular assays and clinical trials will be essential to validate the efficacy and safety of this synbiotic nutraceutical during pregnancy

## 5 Conclusion

The administration of a synbiotic nutraceutical containing the potentially probiotic strains *L. fermentum* 136, *L. fermentum* 263, and *L. fermentum* 296 combined with jabuticaba peel, significantly improved glucose tolerance, reduced low‐grade inflammation, and modulated the GM in rats with GDM. In addition, the results show that synbiotic nutraceutical administration to dams mitigated cardiac autonomic dysfunction in their male and female offspring. These findings suggest the potential of this synbiotic nutraceutical as a therapeutic approach for managing GDM and its associated long‐term complications, although further studies, particularly in humans, are warranted to validate these effects.

## Conflicts of Interest

The authors declare no conflicts of interest.

## Data Availability

The authors have nothing to report.
